# Supplementary figures and images for: Identification of miR-30c-5p microRNA in Serum as a Candidate Biomarker to Diagnose Endometriosis
Source: Int J Mol Sci. 2024 Feb 3;25(3):1853. doi: 10.3390/ijms25031853 (PMC10855247; doi:10.3390/ijms25031853)

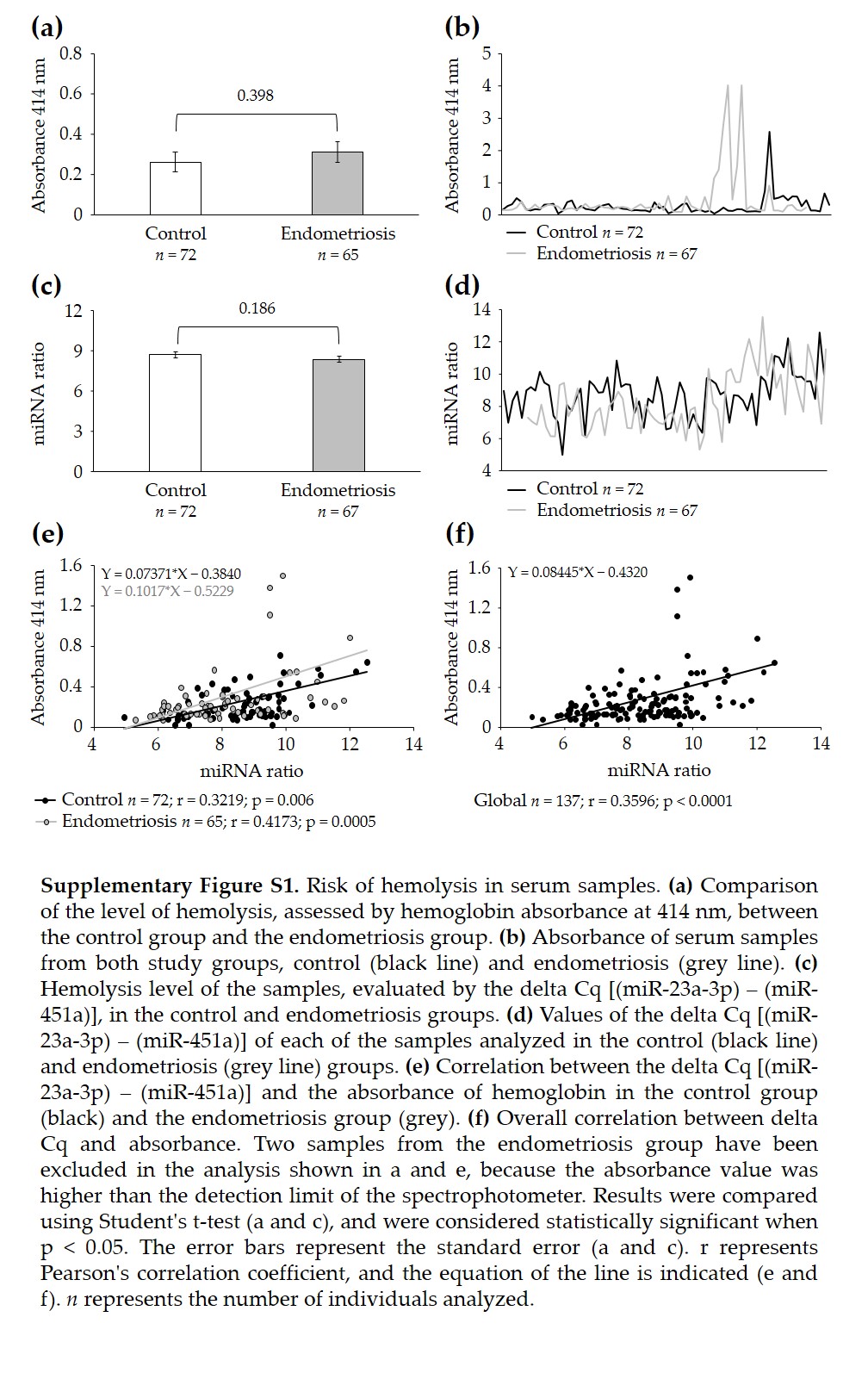

Supplement: Supplementary file 1 [file ijms-25-01853-s001.zip › ijms-2845933-supplementary.jpg]
